# H_2_-induced copper and silver nanoparticle precipitation inside sol-gel silica optical fiber preforms

**DOI:** 10.1186/1556-276X-7-487

**Published:** 2012-08-31

**Authors:** Abdallah Chahadih, Hicham El Hamzaoui, Odile Cristini, Laurent Bigot, Rémy Bernard, Christophe Kinowski, Mohamed Bouazaoui, Bruno Capoen

**Affiliations:** 1 Laboratoire de Physique des Lasers, Atomes et Molécules (CNRS, UMR 8523), IRCICA (USR CNRS 3380), CERLA (FR CNRS 2416), Bâtiment P5, Université Lille 1-Sciences et Technologies, Villeneuve d'Ascq Cedex, 59655, France

**Keywords:** copper nanoparticles, silver nanoparticles, surface plasmon resonance, sol-gel process, dense silica preforms.

## Abstract

Ionic copper- or silver-doped dense silica rods have been prepared by sintering sol-gel porous silica xerogels doped with ionic precursors. The precipitation of Cu or Ag nanoparticles was achieved by heat treatment under hydrogen followed by annealing under air atmosphere. The surface plasmon resonance bands of copper and silver nanoparticles have been clearly observed in the absorption spectra. The spectral positions of these bands were found to depend slightly on the particle size, which could be tuned by varying the annealing conditions. Hence, transmission electron microscopy showed the formation of spherical copper nanoparticles with diameters in the range of 3.3 to 5.6 nm. On the other hand, in the case of silver, both spherical nanoparticles with diameters in the range of 3 to 6 nm and nano-rods were obtained.

## Background

Nanoparticles (NPs) of noble metals, namely gold, copper and silver, have unique optical, electrical, and magnetic properties. It is well known that these properties, and in particular the optical absorption, differ drastically from the properties of bulk metals. The physical origin of the light absorption by metallic NPs is the excitation of coherent oscillations of the conduction band electrons by light, which is known as surface plasmon resonance (SPR) [1]. Recent investigations showed that SPR-based devices have enhanced properties useful for many optoelectronic materials and devices, such as solar cells [2] or emitters [3]. Dense silica matrices, doped by NPs of noble metals, have stimulated considerable interest in basic research as well as from the applied standpoint in connection with enhancement of catalytic behavior [4], sensors [5], linear and nonlinear optical properties [6-9].

The major efforts in the synthesis of metallic nanoparticles inside a silica glass are based on two methods: melting process [8-12] and sol-gel route [7,13,14]. One of the most important features of doped sol-gel silica materials is their ability to preserve chemical and physical properties of the dopants at high temperature. Precipitations of copper and silver NPs in sol-gel silica glasses have been reported by Yeshchenko et al. [13,14]. Examining the synthesis details, one can note that their porous silica matrices were produced by the conventional sol-gel technique. Then, the samples were soaked into an alcoholic solution of copper nitrate or silver nitrate before being densified at 1,200°C under hydrogen atmospheres. These densifications led to the formation of NPs. However, the synthesis of such silica glass doped with noble metal NPs has been limited to powders or thin films. To our best knowledge, the present work reports on the first precipitation of these NPs inside a bulk silica optical preform.

Recently, our research team reported the fabrication of a microstructured optical fiber drawn at about 2,000°C with a sol-gel silica cylindrical rod doped with gold NPs as a fiber core [15]. The gold NPs were precipitated during the sintering process under air. Such optical fibers presented interesting linear and nonlinear optical properties. In this work, we report on the precipitation of copper or silver NPs inside sol-gel silica bulk rods. To this purpose, a reducing thermal processing has been applied after the glass densification. The interest of this method lies in the possible preservation of these NPs inside silica glass preforms even at a high temperature, which is promising for the achievement of silver- or copper-doped optical fiber cores.

## Methods

### Chemical materials

Tetraethyl orthosilicate (TEOS) (≥99%), copper (II) hexafluoroacetylacetonate hydrate, silver (I) hexafluoroacetylacetonate (1,5-cyclooctadiene) and methanol CHROMASOLV were employed for the synthesis of ionic copper-doped and ionic silver-doped silica glasses. All these products were purchased from Sigma-Aldrich Chimie S.a.r.l. (Lyon, France).

### Preparation of samples

**Figure 1:**
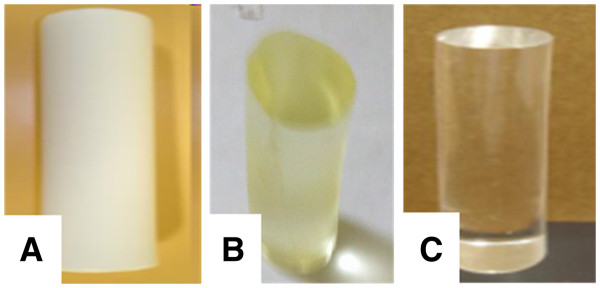
**Photographs of silica glasses.** (**A**) Undoped silica xerogel, (**B**) ionic copper-doped silica glass and (**C**) ionic silver-doped silica glass.

The first step of the fabrication is the synthesis of a cylindrical porous rod by the sol-gel route. This technique has been chosen because it enables to produce transparent glasses at temperatures lower than the ones necessary for the conventional vapor-based processes used in the optical fiber industry. Non-transparent centimeter-length xerogels, shaped as rods (Figure 1A), were obtained from the hydrolysis and condensation of TEOS [16]. These porous rods, exhibiting interconnected nanometric pores (≈25 nm), were impregnated in an alcoholic solution of copper (II) hexafluoroacetylacetonate hydrate or silver (I) hexafluoroacetylacetonate (1,5-cyclooctadiene). Then, the samples were taken out and dried for several hours in order to remove the solvents and to retain the precursor within the pores. The resulting doped xerogels were then densified under air atmosphere at 1,200°C to obtain transparent and cylindrical doped silica glasses (Figure 1B,C). At this stage, no formation of copper or silver NPs has been observed. After densification, these vitreous rods were heated at 80°C under a 140-bar hydrogen atmosphere for 2 weeks. The colors of the samples were kept similar before and after the hydrogenation process. Then, the same hydrogenated samples were heated under air atmosphere at different temperatures ranging between 100°C and 1,200°C for 1 h and 30 min (temperature slope, 10°C/min).

### Sample characterization

Before any characterization, the obtained cylindrical glass rods were cut into pieces of 1-mm thick, which were polished to get high optical transparency. The samples were characterized by absorption spectroscopy and transmission electron microscopy (TEM). The absorption spectra were recorded at room temperature using a Perkin-Elmer Lambda 19 UV-vis-IR double beam spectrometer (MA, USA). The TEM characterization was performed on a microscope FEI Tecnai G2-20 twin with a 200-kV acceleration voltage (FEI Company, Eindhoven, The Netherlands). For TEM imaging, the sample was cut and polished to a 50-μm thickness and deposited on a molybdenum microscope grid. Then, an argon ion gas milling process allowed to obtain a thin area before a carbon film was evaporated on it.

## Results and discussion

### Cu-doped silica glass rod

**Figure 2:**
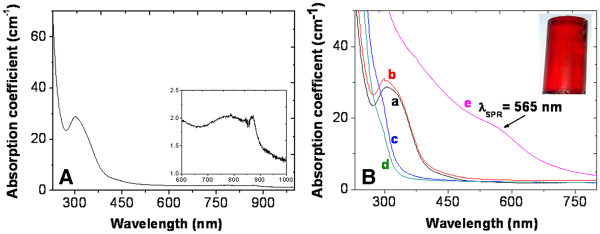
**Optical absorption spectra of dense silica glass doped with the copper precursor.** (**A**) Before any reduction process; inset: zoomed scale near 800 nm. (**B**) After hydrogenation at 80°C without any further heat treatment (curve a) and after annealing at 100°C (curve b), 300°C (curve c), 500°C (curve d) and 700°C (curve e). Inset: a picture of the silica rod heated at 700°C.

Figure 2A presents an optical absorption spectrum of the densified silica glass doped with the copper precursor, which exhibits two absorption bands at 303 and 800 nm. The peak centered at 303 nm is related to Cu^+^ ions stabilized in the silica glass [13], while the weaker and broader band around 800 nm is attributed to Cu^2+^ ions [10]. Moreover, optical absorption spectra have been recorded on the hydrogenated sample and after annealing under air atmosphere at different temperatures. According to Figure 2B, the sample heated at 100°C under air condition shows approximately the same absorption spectrum as the non-annealed sample. On the other hand, a heat treatment at a temperature between 300 and 500°C led to a strong decrease in the absorbance of the Cu^**+**^-related peak at 303 nm likely due to the reduction of Cu^+^ into Cu^0^. The band due to Cu^2+^ centered at 800 nm also disappeared after heating at 300°C. The annealing at 700°C led to the appearance of a red color originating from the growth of copper NPs, as confirmed by the occurrence of the Cu NPs-related SPR band around 565 nm. The homogeneous repartition of this coloration in the whole volume of the sample has been checked by polishing it at different depths.

**Figure 3:**
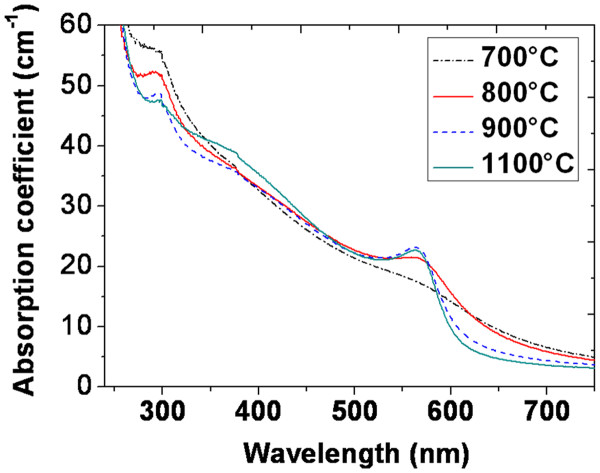
**Absorption spectra of the copper-doped silica after hydrogenation and annealing at different temperatures.** Done for 1 h and 30 min.

When the annealing temperature was further increased (Figure 3), the maximum of the SPR band did not shift in wavelength, but the band became narrower. This behavior can be explained by the increase in the NP size [17,18] caused by an enhanced mobility of the reduced atom inside the silica glass at higher annealing temperature.

The mean diameter of Cu NPs after annealing at each temperature has been evaluated using the following equation (Equation 1) [19,20]:

(1) $$d=V_{\text{F}}/\Delta \omega_{1/2}\text{,}$$

where *d* is the diameter of NPs, *V*_F_ is the Fermi velocity of electrons in bulk copper (1.57 × 10^6^ m/s) and *Δω*_*1/2*_ = *2πc (1/λ*_*1*_ *− 1/λ*_*2*_*)* is the bandwidth at half maximum (FWHM) in frequency of the SPR absorption band. The so-calculated mean average diameter of Cu NPs is summarized in Table 1.

**Table 1:** Calculated diameters of Cu NPs as a function of heating temperature

**Temperature (°C)**	**700**	**800**	**900**	**1,100**
Diameter (nm)	3.3	4.3	5.6	5.6

**Figure 4:**
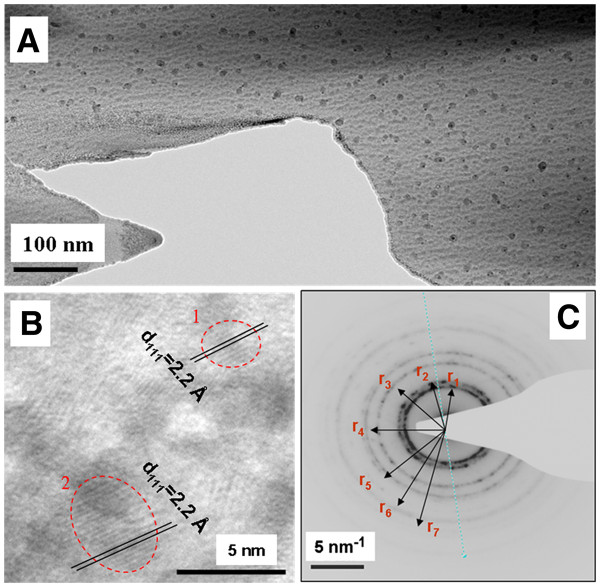
**TEM, HR-TEM and electron diffraction images.** (**A**) TEM image taken from the sample heated at 900°C, (**B**) HR-TEM image of several Cu NPs and (**C**) electron diffraction image taken from a zone filled with NPs.

Figure 4A shows a TEM image taken from the sample heated at 900°C for 1 h and 30 min. Quasi-spherical NPs, with a good spatial distribution and a diameter between 3 and 10 nm, were observed. Figure 4B presents a HR-TEM image of the different NPs. The analysis of the circled zones gives an interplanar distance of 2.2 ± 0.1 Å, which can be attributed to the (111) lattice planes of metallic copper (d_111_ = 2.09 Å; JCPDS card no. 4–0836). In addition, Figure 4C presents an electron diffraction pattern taken in a zone containing several NPs. The estimated diffraction radii from r_1_ to r_7_ correspond well to the Cu lattice planes (111), (200), (220), (311), (222), (400) and (331), respectively. These results reveal the formation of copper NPs inside the silica glass prepared by a sol-gel route.

**Figure 5:**
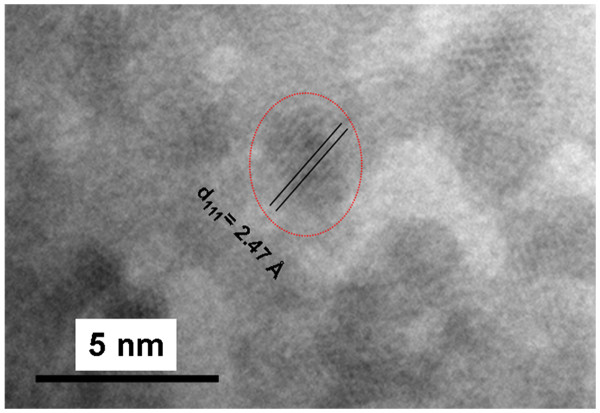
**HR-TEM taken from a set of Cu**_**2**_**O NPs formed inside the silica glass.** Image after heating at 900°C.

On the other hand, copper oxide (Cu_2_O) could also be formed after heating under air atmosphere at 900°C. This oxidation can be expected from the large absorption band centered on 400 nm in Figure 3[13]. Furthermore, the presence of such oxide particles has been confirmed in some HR-TEM images. For instance, in Figure 5, a set of NPs exhibits an interplanar distance corresponding to the [111] direction of Cu_2_O (d_111_ is approximately 2.47 Å; JCPDS card no. 4–0836) .

### Ag-doped silica glass rod

**Figure 6:**
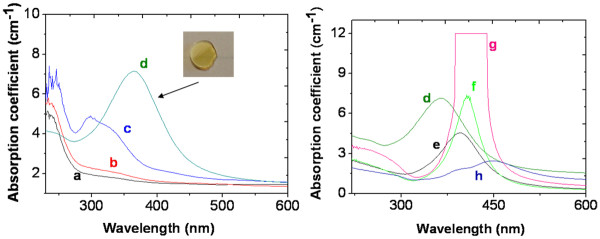
**Optical absorption spectra of a dense silica glass doped with silver.** Before hydrogenation (**a**), after hydrogenation (**b**), after hydrogenation and annealing at 300°C (**c**), 500°C (**d**), 700°C (**e**), 900°C (**f**), 1,100°C (**g**) and 1,200°C (**h**) for 1 h and 30 min. Inset: photograph image of the sample heated under air condition at 500°C for 1 h and 30 min.

The color of the Ag^+^-doped sample after hydrogenation turned yellow after heating at 500°C for 1 h and 30 min, as shown in the inset picture of Figure 6. The homogeneous repartition of this coloration in the whole volume of the sample has been checked by polishing it at different depths.

The absorption spectra of Figure 6, almost identical before and after the hydrogenation process, exhibit a band at 230 nm attributed to silver ions [21] and a smooth shoulder around 330 nm, corresponding to atomic Ag^0^[21]. Heating the sample at 100°C under air condition did not change the spectrum, but from an annealing temperature of 300°C up to 1,100°C, the peak at 330 nm became more intense, revealing a much larger amount of reduced species. Heating at 500°C leads to the aggregation of the silver atoms into small clusters, as indicated by the presence of a silver SPR at 367 nm, which is considerably blue-shifted with regard to the normal position of the SPR band in silica (410 nm) [14]. This conventional position (410 nm) is attained when the temperature is increased up to 700°C, as shown in Figure 6e. Moreover, the absorbance of the SPR band strongly increased and was slightly red-shifted with the heating temperature up to 1,100°C. The maximum NP concentration is attained after heat treatment at 1,100°C, as attested by the saturation of the spectrophotometer. On the contrary, after annealing at 1,200°C, the SPR band decreased probably due to oxidation of Ag NPs. Meanwhile, for this latter annealing temperature, the SPR band was divided into two sub-bands, the first one being centered at 390 nm and the second one at 450 nm. The appearance of these two peaks could be explained by the formation of elongated shapes of silver NPs, such as nanorods [22]. Equation 1 was used to roughly estimate the mean particle size of NPs from the SPR peak width after annealing at 500°C, 700°C and 900°C, yielding diameters of 1, 1.6 and 2.7 nm, respectively.

**Figure 7:**
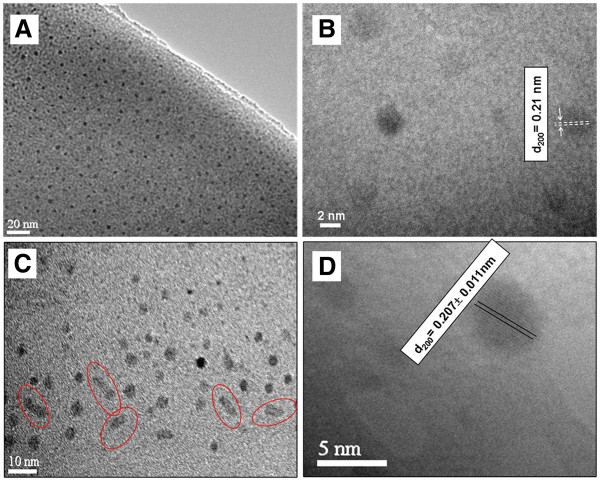
**TEM measurements performed on hydrogenated dense silica doped with silver.** Heated at 1,100°C (**A**, **B**) and 1,200°C (**C**, **D**).

**Figure 8:**
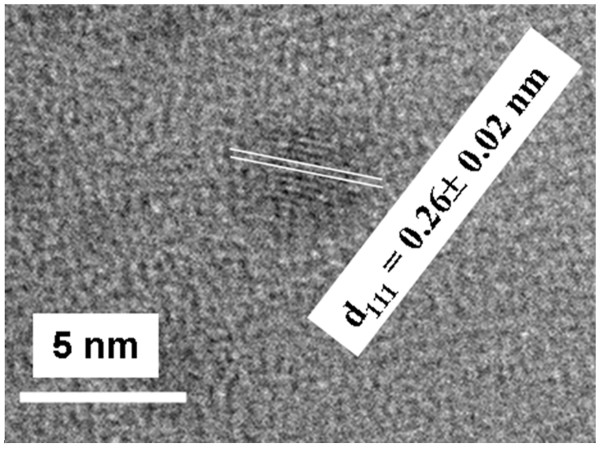
HR-TEM image taken from the hydrogenated sample post-heated at 1,200°C.

To confirm the formation of silver NPs and to explain the appearance of these two SPR positions after heating at 1,200°C, TEM measurements were performed on the samples heated at 1,100°C and at 1,200°C (Figure 7). Figure 7A,B shows TEM images of the sample heated at 1,100°C for 1 h and 30 min. Quasi-spherical NPs, with a size distribution ranging between 2 and 5 nm in diameter, were observed. The atomic interplanar distance, as measured in the HR-TEM image of Figure 7B, is around 0.21 nm which is attributed to the (200) lattice planes of cubic Ag (d_200_ = 0.2044 nm; JCPDS cards 4–0783). In Figure 7C, the TEM image of the sample heated at 1,200°C is presented: spherical and non-spherical NPs with a size distribution ranging between 3 and 10 nm were observed. The presence of small rods, with an aspect ratio larger than 2, could indeed explain the two SPR bands in the absorption spectrum at this temperature. In the HR-TEM image of a single NP (Figure 7D), the observed lattice planes (200) of Ag metal can be once again identified. To the best of our knowledge, this is the first report of such nano-rods in a dense pure silica glass. Moreover, the sudden and strong decrease of the SPR band after heat treatment at 1,200°C (Figure 6h) is coherently ascribable to silver oxidation. In effect, Figure 8 presents a HR-TEM image of a single oxide NP, where the (111) lattice planes of Ag_2_O could be identified (d_111_ = 0.273 nm; JCPDS cards 4–0783).

### Mechanism of NP formation

The formation mechanisms of Cu or Ag NPs inside a dense silica rod where H_2_ molecules have diffused could be explained by the following reactions [23,24]:

(2) ≡Si-O−Cu++12H2→≡Si-OH+Cu0

(3) $${\text{nCu}}^{0}\;\overset{\Delta >700{}^{\circ}\text{C}}{\to }{\;\text{(Cu})}_{\text{n}}$$

(4) ≡Si-O−Ag++12H2→≡Si-OH+Ag0

(5) $${\text{nAg}}^{0}\overset{\Delta >500{}^{\circ}\text{C}}{\to }{\left(\text{Ag}\right)}_{\text{n}}$$

The red or yellow color observed inside the volume of the dense silica rods indicates that hydrogen molecules are allowed to penetrate the matrix at 80°C under a pressure of 140 bars in order to reduce the metal ions. Nevertheless, the diffusion of hydrogen inside the silica glass, i.e., near the copper or silver ions, is not sufficient to reduce them. To that purpose, the hydrogenated sample should be subsequently annealed at a temperature which depends on the ion. From this point of view, Cu^+^ ions are more difficultly reduced (annealing temperature of 700°C required) than Ag^+^ ions (temperature of 500°C is required).

## Conclusion

Finally, NPs of noble metals were precipitated in silica preforms prepared by the sol-gel process. The method consists of a heat treatment of the densified Cu- or Ag-doped glasses under hydrogen atmosphere, followed by an annealing in air atmosphere. It has been shown that the Cu NP size increased as a function of this last annealing temperature, and the obtained diameters ranged between 3 and 6 nm. Similarly, it has been shown that the control of the annealing temperature allows the adjustment of both size and shape of the Ag NPs. The formation of both copper oxide (Cu_2_O) and silver oxide (Ag_2_O) NPs at high temperature has also been confirmed by TEM analysis.

The preservation of metal NPs at temperatures as high as 1,100°C is very promising. In the next future, each of these Cu or Ag cylindrical rods will be drawn into a small capillary and then stacked with pure silica tubes in order to get a holey preform.

## Abbreviations

MOF, microstructured optical fiber; NP, nanoparticle; TEM, transmission electron microscopy; TEOS, tetraethoxysilane; SPR, surface plasmon resonance.

## Competing interests

The authors declare that they have no competing interests.

## Authors' contributions

AC (doctor), HEH (engineer) and BC (professor) designed, analyzed, and performed the experiments and wrote this report. MB (professor) is responsible for the correction of this report. AC (doctor), HEH (engineer), OC (assistant professor), RB (assistant professor), LB (researcher), CK (assistant professor), BC (professor) and MB (professor) have performed the interpretation of results as well as the draft of the manuscript, and gave the final approval of the version to be published. All authors read and approved the final manuscript.

## References

[B1] Kelly KL Coronado E Zhao LL Schatz GC The optical properties of metal nanoparticles: the influence of size, shape, and dielectric environment J Phys Chem B 2003 107 668 677

[B2] Wu J Mangham SC Reddy VR Manasreh MO Weaver BD Surface plasmon enhanced intermediate band based quantum dots solar cell Solar Energy Materials & Solar Cells 2012 102 44 49

[B3] Wu J Lee S Reddy VR Manasreh MO Weaver BD Yakes MK Furrow CS Kunets VP Benamara M Salamo GJ Photoluminescence plasmonic enhancement in InAs quantum dots coupled to gold nanoparticles Mater Lett 2011 65 3605 3608 10.1016/j.matlet.2011.08.019

[B4] Liu S Han MY Silica-coated metal nanoparticles Chem Asian J 2010 5 36 45 19768718 10.1002/asia.200900228

[B5] Hu J Wang Z Li J Gold nanoparticles with special shapes: controlled synthesis, surface-enhanced Raman scattering, and the application in biodetection Sensors 2007 7 3299 3311 10.3390/s7123299 PMC3841896 28903295

[B6] Kiran PP De G Rao DN Nonlinear optical properties of copper and silver nanoclusters in SiO2 sol–gel films IEE P-Circ Dev Syst 2003 150 559 562 10.1049/ip-cds:20031131

[B7] Li W Seal S Megan E Ramsdell J Scammon K Lelong G Lachal L Richardson KA Physical and optical properties of sol–gel nano-silver doped silica film on glass substrate as a function of heat-treatment temperature J Appl Phys 2003 93 9553 9561 10.1063/1.1571215

[B8] Strohhofer C Polman A Silver as a sensitizer for erbium Appl Phys Lett 2002 81 1414 1416 10.1063/1.1499509

[B9] Speranza G Minati L Chiasera A Ferrari M Righini GC Ischia G Quantum confinement and matrix effects in silver exchanged sodalime glasses J Phys Chem C 2009 113 4445 4450

[B10] João CS Coelho MP Ruivo A Pires de Matos A Infrared nanosecond laser effects on the formation of copper nanoparticles Mater Lett 2010 64 705 707 10.1016/j.matlet.2009.12.044

[B11] Borsella E Del Vecchio A Garcia MA Sada C Gonella F Polloni R Quaranta A Van Wilderen LJGW Copper doping of silicate glasses by the ion-exchange technique: A photoluminescence spectroscopy study J Appl Phys 2002 91 90 98 10.1063/1.1421241

[B12] Guilherme K Hayakawa T Nogami M Copper reduction and hydroxyl formation by hydrogen process in alumino-silicate glasses J Phys Chem Solids 2011 72 151 157 10.1016/j.jpcs.2010.10.085

[B13] Yeshchenko AO Dmitruk IM Dmytrukb AM Alexeenko AA Influence of annealing conditions on size and optical properties of copper nanoparticles embedded in silica matrix Mat Sci Eng B 2007 137 247 254 10.1016/j.mseb.2006.11.030

[B14] Yeshchenko AO Dmitruk IM Dmytrukb AM Alexeenko AA Losytskyy MY Kotko AV Pinchuk AO Size-dependent surface-plasmon-enhanced photoluminescence from silver nanoparticles embedded in silica Phys Rev B 2009 79 235438

[B15] Bigot L El Hamzaoui H Le Rouge A Chassagneux F Bouwmans G Capoen B Bouazaoui M Linear and nonlinear optical properties of gold nanoparticle-doped photonic crystal fiber Opt Exp 2011 19 19061 19066 10.1364/OE.19.019061 21996846

[B16] El Hamzaoui H Courtheoux L Nguyen V Berrier E Favre A Bigot L Bouazaoui M Capoen B From porous silica xerogels to bulk optical glasses: the control of densification Mat Chem Phys 2010 121 83 88 10.1016/j.matchemphys.2009.12.043

[B17] Kreibig U Vollmer M Optical Properties of Metal Clusters 1995 Springer, New York

[B18] Xiong L Weiping C Huijuan B Optical absorption of copper nanoparticles dispersed within pores of monolithic mesoporous silica J Mater Res 2002 17 1125 1128 10.1557/JMR.2002.0166

[B19] Sheng J Zhang J Qiao L Long-term stability of X-ray induced color centers in silver-doped glass J Non-Cryst Solids 2006 352 2914 2916 10.1016/j.jnoncrysol.2006.04.017

[B20] Pal U Bautista HA Rodriguez-Fernandez L Cheang-Wong JC Effect of thermal annealing on the optical properties of high-energy Cu implanted silica glass J Non-Cryst Solids 2000 275 65 71 10.1016/S0022-3093(00)00246-5

[B21] Ershov BG Janata E Henglein A Fojtik A Silver atoms and clusters in aqueous solution: absorption spectra and the particle growth in the absence of stabilizing Ag+ ions J Phys Chem 1993 97 4589 4594 10.1021/j100120a006

[B22] Lu Y Wang Y Chen W Silver nanorods for oxygen reduction: strong effects of protecting ligand on the electrocatalytic activity J Power Sources 2011 196 3033 3038 10.1016/j.jpowsour.2010.11.119

[B23] Mohr C Dubiel M Hofmeister H Formation of silver particles and periodic precipitate layers in silicate glass induced by thermally assisted hydrogen permeation J Phys Condens Matter 2001 13 525 536 10.1088/0953-8984/13/3/312

[B24] Smedskjaer MM Deubener J Yue Y Inward cationic diffusion and formation of silica-rich surface nanolayer of glass Chem Mater 2009 21 1242 1247 10.1021/cm802513r

